# CSF1R blockade slows the progression of amyotrophic lateral sclerosis by reducing microgliosis and invasion of macrophages into peripheral nerves

**DOI:** 10.1038/srep25663

**Published:** 2016-05-13

**Authors:** Anna Martínez-Muriana, Renzo Mancuso, Isaac Francos-Quijorna, Adrian Olmos-Alonso, Rosario Osta, V. Hugh Perry, Xavier Navarro, Diego Gomez-Nicola, Ruben López-Vales

**Affiliations:** 1 Institut de Neurociencies, Department Biologia Cellular, Fisiologia i Immunologia, Universitat Autònoma de Barcelona, and Centro de Investigación Biomédica en Red sobre Enfermedades Neurodegenerativas (CIBERNED), Bellaterra, Spain; 2 Centre for Biological Sciences, University of Southampton, Southampton General Hospital, Tremona Road, Southampton, SO16 6YD United Kingdom; 3 Laboratory of Genetic Biochemistry (LAGENBIO), Health Research Institute of Aragón, Universidad de Zaragoza, Zaragoza, Spain

## Abstract

Inflammation is a common neuropathological feature in several neurological disorders, including amyotrophic lateral sclerosis (ALS). We have studied the contribution of CSF1R signalling to inflammation in ALS, as a pathway previously reported to control the expansion and activation of microglial cells. We found that microglial cell proliferation in the spinal cord of SOD1^G93A^ transgenic mice correlates with the expression of CSF1R and its ligand CSF1. Administration of GW2580, a selective CSF1R inhibitor, reduced microglial cell proliferation in SOD1^G93A^ mice, indicating the importance of CSF1-CSF1R signalling in microgliosis in ALS. Moreover, GW2580 treatment slowed disease progression, attenuated motoneuron cell death and extended survival of SOD1^G93A^ mice. Electrophysiological assessment revealed that GW2580 treatment protected skeletal muscle from denervation prior to its effects on microglial cells. We found that macrophages invaded the peripheral nerve of ALS mice before CSF1R-induced microgliosis occurred. Interestingly, treatment with GW2580 attenuated the influx of macrophages into the nerve, which was partly caused by the monocytopenia induced by CSF1R inhibition. Overall, our findings provide evidence that CSF1R signalling regulates inflammation in the central and peripheral nervous system in ALS, supporting therapeutic targeting of CSF1R in this disease.

Amyotrophic lateral sclerosis (ALS) is a fatal neurodegenerative disease caused by the loss of motoneurons (MNs) in the motor cortex, brainstem and spinal cord. It manifests with skeletal muscle weakness, spasticity and eventual paralysis, leading to the death of patients by respiratory failure 3 to 5 years after diagnosis^1^. ALS occurs sporadically in 90% of cases whereas the remaining 10% arises from inherited forms of the disease.

In the last decades, the generation of different ALS murine models has allowed the identification of several mechanisms leading to MNs death. The exact pathogenic process that triggers MN degeneration in ALS is currently unknown, but it is likely to be multifactorial, as for other neurodegenerative diseases^2^.

One of the hallmarks of chronic neurodegenerative diseases, including ALS, is the contribution of non-neuronal cells to the progression of the pathology, especially those regulating the neuroimmune component^3^. Several studies indicate that astrocytes are harmful in ALS^4^,^5^, whereas the role of microglial cells is unclear^6^,^7^,^8^,^9^. However, a recent report supports a detrimental role of microglia to the pathology of ALS, inducing MN death via NFκB activation^7^. Besides glial cells, peripheral leukocytes also contribute to ALS^10^,^11^,^12^. Evidence suggests that lymphocytes subsets contribute to slowing disease progression^13^, whereas the function of macrophages, which invade the peripheral nerves during disease progression^14^,^15^, is yet to be defined.

Colony-stimulating factor 1 receptor (CSF1R) is the cell surface receptor for IL-34 and CSF1. CSF1R has important roles in haematopoiesis, regulation of proliferation, cell survival and maturation of microglia and monocytes, as well as in controlling the overall immune response^16^. Recent evidence from a mouse model of prion disease supports that CSF1R controls microgliosis and contributes to neurodegeneration^17^. A previous study showed that systemic administration of CSF1 accelerates disease progression in the SOD1^G37R^ mouse, suggesting that overactivation of CSF1R exerts detrimental actions in ALS, probably, by increasing the mitogenic activity of microglia^18^,^19^. It is important to establish whether pathological activation of CSF1R in ALS contributes to disease progression, and if so, which are the physiological mechanisms underlying its harmful effects, since CSF1 is increased in the spinal cord of ALS patients^19^.

In the present study, we pharmacologically inhibited the activation of CSF1R to dissect the role of this receptor in ALS. We provide evidence that blocking CSF1R ameliorates the clinical course of ALS disease by reducing both the invasion of macrophages into peripheral nerves at pre-symptomatic stages of the disease, and by impeding microglia proliferation at late stages of the pathology.

## Materials and Methods

### Animals

All the experimental procedures were approved by the Universitat Autònoma de Barcelona Animal Experimentation Ethical Committee and followed the European Communities Council Directive 2010/63/EU, and the methods for each procedure were carried out in accordance with the approved guidelines. Experiments were performed in female transgenic mice carrying the G93A human SOD1 mutation (B6SJL-Tg[SOD1-G93A]1Gur) obtained from the Jackson Laboratory (Bar Harbor, ME, USA) and provided from the colony maintained at the University of Zaragoza. Hemizygous transgenic mice were identified by PCR amplification of DNA extracted from tail samples and then were maintained in local facilities. Mice were housed with food and water ad libitum at room temperature of 22 ± 2 °C under a 12:12-h light–dark cycle. It was considered that animals reached the endpoint of the disease when the righting reflex was lost for longer than 30 s.

At 8 weeks of age (prior to the beginning of the treatment), animals were electrophysiologically tested to obtain baseline levels. Animals were then distributed among the different experimental groups according to their progenitors, weight and electrophysiology baseline values in balanced groups, either GW2580-treated or untreated control SOD1^G93A^ mice. Inhibition of CSF1R was achieved by administration of GW2580 as described previously^17^. Briefly, GW2580 (LC Laboratories) was dissolved in sterile 0.9% saline buffer with 0.1% tween80, and administered by oral gavage providing a daily dose of 75 mg/kg from 8 weeks of age until the end of the experiment. When required (assessment of microglia proliferation), mice received intraperitoneal injections of EdU (Invitrogen; 0.1 mg/kg in 0.2 ml of sterile PBS) daily for the 5 days before the end of the experiment.

### Functional tests

Motor coordination, balance and strength of the animals were assessed using the Rotarod test^20^. All mice were trained three times a week on the rod rotating at constant speed of 14 rpm (rotating cylinder 3.4 cm diameter) for a maximum of 180 seconds to reach the baseline level of performance. Animals were then tested weekly from 8 until 16 weeks of age at the same speed, and the time for which each animal could remain on the rotating rod was measured. An arbitrary maximum time of remaining on the rotating rod of 180 s was considered (n = 10 per group).

The highest locomotion speed of the mice was tested on a controlled treadmill at the end of the follow up (16 weeks of age). Mice were placed over the treadmill and their capacity to run with increasing treadmill velocities, 5, 10, 15, 20, 25 and 30 cm/s, was recorded^21^ (n = 10 per group).

Motor nerve conduction tests were performed every 2 weeks from 8 weeks to 16 weeks of age (n = 18 SOD1^G93A^ control, n = 19 SODG^G93A^ treated with GW2580). The sciatic nerve was percutaneously stimulated through a pair of needle electrodes placed at the sciatic notch, by means of single pulses of 0.02 ms duration (Grass S88). The compound muscle action potential (CMAP, M-wave) was recorded from the tibialis anterior (TA) and the plantar interossei (PL) muscles with microneedle electrodes^21^. All potentials were amplified and displayed on a digital oscilloscope (Tektronix 450S) at settings appropriate to measure the amplitude from baseline to the maximal negative peak and the latency from stimulus to the onset of the first negative deflection, to the maximal negative peak and to the end of the wave. The recording needles were placed using a microscope and guided by anatomical landmarks, to ensure reproducibility of needle location on all animals. During the tests, the mouse skin temperature was maintained between 34 and 36 °C using a thermostatically controlled heating pad. All the evaluators were blinded to the experimental groups.

### Histology

At 16 weeks of age, female SOD1^G93A^ GW2580-treated (n = 9), SOD1^G93A^ untreated (n = 7) and age-matched WT littermates (n = 7) were transcardially perfused with 4% paraformaldehyde (PFA) in 0.1 M phosphate buffer (PB). Lumbar segments of the spinal cords were harvested, post-fixed in 4% PFA for 2 hours and cryoprotected with 30% sucrose in 0.1 M PB at 4 °C. Samples were cut in transverse serial sections (40 μm thick) with a cryostat (Leica) between L2 and L6 segments. For each segment, series of 10 sections were sequentially collected on free-floating and kept in Olmos solution at −20 °C. To analyse MNs preservation, sections were rehydrated for 1 min and stained for 2 h with an acidified solution of 3.1 mM cresyl violet. Sections were then washed in distilled H20 for 1 min, dehydrated and mounted with DPX (Fluka). MNs were identified by their localization in the ventral horn of the stained spinal cord sections and quantified following size and morphological criteria: only MNs with diameters larger than 20 μm and with polygonal shape and prominent nucleoli were quantified. MNs present in the lateral site of both ventral horns were quantified in four serial sections of the L4 segment^21^. Spinal cord tissue sections were also immunostained with rabbit anti-Iba1 (1:500, Wako), goat anti-Iba1 (1:250, Abcam), rat anti-MCH II (1/500 Ebioscience) or rabbit anti-PU.1 (1/500 Cell Signalling) and rabbit anti-CSF1R (1:100, Santa Cruz) primary antibodies for visualization of microglia, and detected with appropriate secondary antibodies conjugated with Alexa 488, 594 (Life Technologies) for immunofluorescence, or biotin for light microscopy (1:200, Vector Labs). For visualization of proliferative microglia, EdU was visualized using the Click-iT reaction coupled to an Alexa Fluor 568 azide following the instructions of the manufacturer (Invitrogen). Nuclei were visualized by DAPI staining and the sections were mounted with Mowiol/DABCO (Sigma-Aldrich) mixture. Quantification of Iba1 and MHCII cells was done as we reported previously^17^,^22^.

Tibial nerves from female WT, treated and untreated SOD1^G93A^ mice were harvested at 12 and 16 weeks of age (n = 4 per group and time point). Nerve samples were cut in longitudinal serial sections of 15 μm thickness with a cryostat. Macrophages were detected by immunostaining using rat anti-mouse F4/80 antibody (1:150, Serotec) as described above. Similarly, a segment of L5 spinal nerve containing the dorsal root ganglia, as well as, the dorsal and ventral roots was removed from female untreated SOD1^G93A^ mice (n = 4), cut in longitudinal sections of 15 μm thickness with a cryostat, and stained against F4/80 for assessing the presence of macrophages in the dorsal and ventral roots. Macrophages were manually quantified in a 7.5 × 10^4^ μm^2^ area from 4 tissue sections of tibial nerve, sciatic nerve and dorsal and ventral roots.

Tissue sections were viewed with an Olympus BX51 microscope and images were captured using an attached Olympus DP50 digital camera, using the Cell^A Image acquisition software. Alternatively, sections were visualized in a Leica CTR 5000 microscope, coupled to a Leica DFC300FX microscope camera or in a Zeiss LSM 700 confocal microscope. Data was represented as number of positive cells/mm^2^. All quantifications were performed blinded to the experimental groups with the help of the ImageJ image analysis software (NIH).

### Fluorescent activated cell sorting (FACS) analysis

At 10, 12 and 16 weeks of age, cells from lumbar spinal cord of SOD1^G93A^ GW2580-treated (n = 4 per time point), SOD1^G93A^ untreated mice (n = 4 per time point) and age-matched WT littermates (n = 4; 16 weeks) were analysed by FACS analysis. Mice were terminally anesthetized with an overdose of sodium pentobarbital and transcardially perfused with 0.9% saline solution. Spinal cord lumbar segments were harvested, mechanically triturated and then passed through a cell strainer of 40 μm mesh (BD2 Falcon) with DMEM media with 10% of Fetal Bovine Serum (FBS). The cell suspension was centrifuged twice at 300 g for 10 min at 4 °C, to remove debris.

Samples were split in several tubes and immunostained. Primary antibody labelling was performed for 1 hour at 4 °C, using DMEM + 10% FBS as buffer. Cells were labelled with the following antibodies: PE or APC Cy7-conjugated anti-CD11b (1:100, eBioscience), PerCP or PerCP-Cy5 conjugated anti-CD45 (1:100, Biolegend), APC conjugated anti-CD86 (1:100, BD Biosciencies), APC conjugated anti-F4/80 (1:100, eBioscience), FITC conjugated anti-iNOS (1:100, BD Biosciences), FITC conjugated anti-CD206 (1:100, Biolegend), PE conjugated anti-CD16/CD32 (1:100, Biolegend) and goat anti-Arginase 1 (1:100, Santa Cruz) followed by secondary incubation with PE conjugated anti-goat (1:150, Abcam). For intracellular analysis of EdU, cells were fixed, washed, permeabilized and incubated with Alexa Fluor 488 conjugated anti-EdU following the Click-iT reaction according to the manufacturer’s instructions (1:100, Life Technologies). Moreover, unstained cells and isotype-matched control samples were used to control for autofluorescence and/or non-specific binding of antibodies. Microglial cells were identified as CD45^low^, CD11b^ + ^and further differentiated based on CD86, iNOS, CD16/32, Arg1 and CD206 expression relative to their activation state.

Sciatic nerve samples from untreated SOD1^G93A^ (n = 3) were also harvested at 16 weeks and processed as described above. Nerve macrophages (CD45^+^, CD11b^+^, F4/80^+^ cells) were further differentiated based on iNOS, CD16/32, Arg1 and CD206 expression.

In addition, blood samples from 12 weeks old SOD1^G93A^ mice untreated or treated with GW2580 (n = 4 per group) were collected from the peroneal vein, erythrocytes were then lysed in RBC lysis buffer (eBioscience), and leukocytes were stained with PECy7-conjugated anti-CD11b (1:100, eBioscience), PerCP or PerCP-Cy5 conjugated anti-CD45 (1:100, Biolegend), APC conjugated anti-F4/80 (1:100, eBioscience) using the same method described above. Monocytes were identified as CD45^+^, CD11b^+^ and F4/80^+^. Cells were analysed in a blinded fashion with respect to the experimental groups using FlowJo^®^ software on a FACSCanto flow cytometer (BD Biosciences).

### Isolation of microglia from CNS tissue

Briefly, spinal cord and brain from adult C57/Bl6 mice (8–10 weeks old) were removed and enzymatically digested with a collagenase B 0.2% (Roche Diagnostics GmbH) and trypsine-EDTA 0.2% at 37 °C for 30 min, and then passed through a cell strainer of 40 μm mesh (BD falcon). Cell suspension was centrifuged twice at 300 g for 10 minutes at 4 °C, and microglial cells were first isolated by magnetic sorting using a CD11b antibody (MiltenyiBiotec) and then stained with PerCP-Cy5.5-conjugated CD45 and PE-Cy7-conjugated CD11b antibodies for further purification on cell sorter (FACSARIATM III, BD Bioscience). Microglia cells were assessed on a flow cytometer (FACSCalibur; BD Biosciences), and only populations presenting >90% purity were used for gene expression analysis.

### Analysis of gene expression by qPCR

At 12 and 16 weeks of age, SOD1^G93A^ GW2580-treated (n = 4 per time point), SOD1^G93A^ untreated mice (n = 4 per time point), and 16 weeks of age WT littermates (n = 3) were processed to obtain samples from the lumbar spinal cord. RNA from spinal cords and *in vivo* sorted microglia was extracted using the RNAqueous^®^-Micro Kit (Life Technologies), quantified using Nanodrop (Thermo Scientific), to be retro-transcribed using the iScript cDNA Synthesis Kit (Bio-Rad), following manufacturer’s instructions, after checking its integrity by electrophoresis in a 2% agarose gel. cDNA libraries were analysed by qPCR using the iTaq Universal SYBR Green supermix (Bio-Rad) and the following custom designed gene-specific primers (Sigma-Aldrich)^17^.

Quality of the primers and qPCR reaction were evaluated by electrophoresis in a 1.5% agarose gel, checking the PCR product size. Data was analysed using the 2-∆∆Ct method with Primer Opticon 3 software, using GAPDH as housekeeping gene.

### Multiplex Assay

At 12 and 16 weeks of age, lumbar spinal cord of SOD1^G93A^ GW2580-treated (n = 4 per time point), SOD1^G93A^ untreated mice (n = 4 per time point), and WT littermates (n = 3; age 16 weeks) were homogenized and after centrifugation at 13000G for five minutes, supernatants were used for cytokine analysis. Protein levels of 11 cytokines (CSF1, TNF-α IL-1α, IL-1β, IL-4, IL-6, IL-10, IL-13, IP-10, KC, MCP-1 and MIP1α.) were assessed using a MILLIPLEX^®^ MAPmagnetic bead-based multi-analyte panel Multiplex bead kit (Merck-Millipore). Standard curves were generated using the specifics standards supplied by the manufacturer. Samples were analysed on a MAGPIX^®^ system (Millipore) using the MILLIPLEX^®^ Analyst 5.1 software (Millipore).

### Statistical analysis

Data are shown as mean ± SEM and analysed using the GraphPad Prism 6 software package (GraphPad Software). Electrophysiological and locomotion test results were analysed using two-way repeated measurements ANOVA with Tukey post-hoc test for multiple comparisons. T-Student was used for histological data comparing the 2 experimental groups. Two-way ANOVA was used for the gene expression data, FACS analysis and multiplex data, followed by a Tukey post-hoc test for multiple comparisons. Survival data was analysed using the Mantel-Cox test. Differences were considered significant at p < 0.05.

## Results

### Characterization of the components of the CSF1R pathway in the spinal cord of SOD1^G93A^ mice

**Figure 1:**
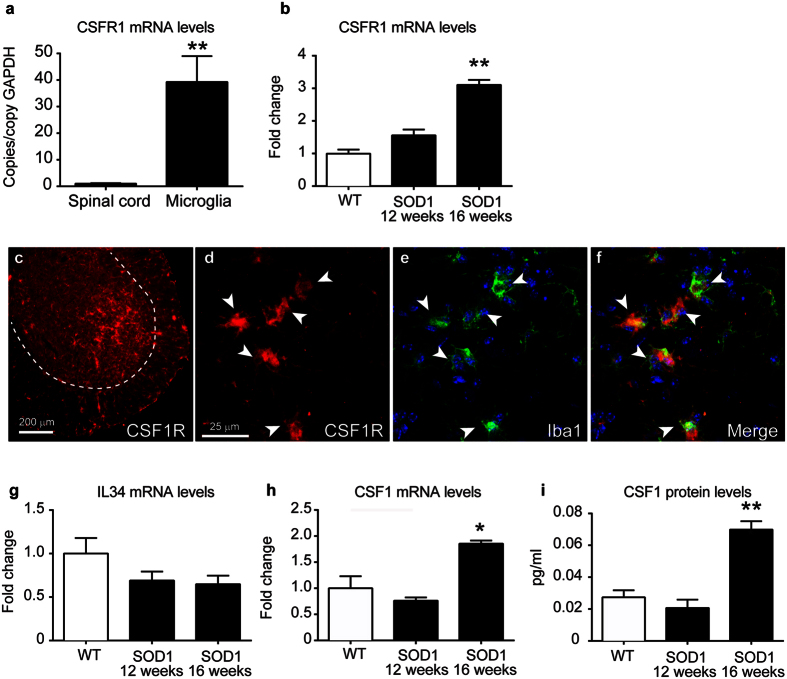
Characterization of the CSF1R pathway in the spinal cord of SOD1^G93A^ mice. (**a**) Analysis of the mRNA expression of CSF1R in the spinal cord and microglial cells sorted from adult CNS of C57/Bl6 mice (n = 4 per group). Note that CSF1R expression is highly enriched in sorted microglia as compared to spinal cord homogenates. (**b**) Expression of CSF1R in the ventral spinal cord of WT and SOD1^G93A^ mice at symptomatic (12 weeks) and end-stage (16 weeks) of the disease (n = 3 in WT, n = 4 in SOD1^G93A^ per time point). (**c**–**f**) Representative immunofluorescence image of CSF1R of the lumbar spinal cord of SOD1^G93A^ mice at 16 weeks of age (**c**) showing that CSF1R immunoreactivity is markedly increased in the ventral horn. Confocal high magnification images of the ventral horn showing the expression of CSF1R (**d**) and Iba1 (**d**). Note in the merged image (**f**) that microglial cell are the source of CSF1R in the SOD1^G93A^ mice at end stage of the disease. Analysis of mRNA levels of IL-34 (**g**) and CSF1 (**h**) in the ventral lumbar spinal cord of WT and SOD1^G93A^ at symptomatic (12 weeks) and end-stage (16 weeks) of the disease (n = 3 in WT, n = 4 in SOD1^G93A^ per time point). (**i**) Quantification of the protein levels of CSF1 in spinal cord homogenates of WT and SOD1^G93A^ mice at 12 and 16 weeks. (n = 3 in WT, n = 4 in SOD1^G93A^ per time point). Note that mRNA and protein levels of CSF1 are up-regulated in the spinal cord of SOD1^G93A^ mice at the end stage of the disease, but not earlier. *p < 0.05 and **p < 0.01 vs. WT. Error bars indicate SEM.

Microgliosis is a major hallmark of the pathology in diverse neurodegenerative diseases, including ALS. Previous studies have highlighted the importance of CSF1R in microglial proliferation^17^,^18^. We therefore characterized the expression of the components of the CSF1R pathway in SOD1^G93A^ mice. We found that spinal cords and sorted microglial cells from C57Bl/6 mice have constitutive mRNA expression of CSF1R. CSF1R transcripts were enriched 39-fold in microglia as compared to total spinal cord homogenates suggesting that microglial cells are the main source, if not the unique, of CSF1R in the CNS (Fig. 1a). Interestingly, we found that mRNA levels of CSF1R increased in the spinal cord of SOD1^G93A^ mice at late stages of the disease (Fig. 1b). At this time point, histological images reveal that CSF1R expression was markedly increased in the ventral horn of the spinal cord (Fig. 1c). Confocal microscopy revealed that reactive microglia was source of the increased expression of CSF1R in this ALS mouse model (Fig. 1d–f). Analysing the expression of the CSF1R ligands, we observed that the mRNA levels of IL-34 remained unaltered in the lumbar spinal cord of SOD1^G93A^ mice, whereas the transcripts for CSF1 increased ~2 fold but only at late stages of the disease (16 weeks) (Fig. 1g,h). This data correlated with the measurement of the protein levels of CSF1 at the lumbar spinal cord of SOD1^G93A^ mice, with upregulated levels at 16 weeks when compared to WT littermates (Fig. 1i). Overall, these findings show that the activation of CSF1R signalling via CSF1 occurs in the spinal cord of SOD1^G93A^ mice at late stages of the disease.

### Inhibition of CSF1R reduces microglial proliferation in SOD1^G93A^ mice

**Figure 2:**
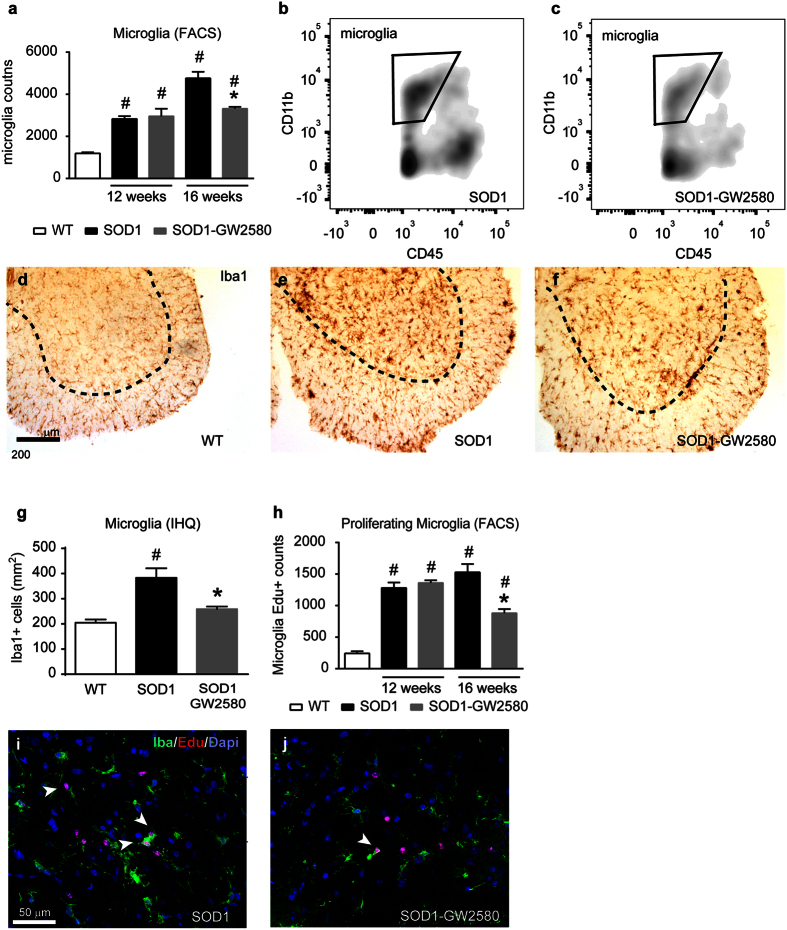
Assessment of microglial cells in lumbar spinal cord of SOD1^G93A^ mice. (**a**–**c**) FACS analysis from lumbar spinal cord of WT, SOD1^G93A^ untreated and treated with GW2580 showing microglial cell counts (CD45low, CD11b+) at 12 and 16 weeks. (n = 4 animals per group and time point). (**b**,**c**) Representative density FACS plots showing the reduction of microglia in 16 weeks old SOD1^G93A^ mice after GW2580 treatment. (**d**–**f**) Representative micrographs of L4-L5 ventral horns stained for Iba1 from (**d**) WT, (**e**) SOD1 control or (**f**) GW2580-treated SOD1 mice. (**g**) Graph showing immunohistochemical quantification of Iba1+ cells in the ventral horns of spinal cord tissue sections of 16 weeks of age SOD1^G93A^ mice untreated or treated with GW2580 (n = 4 per group). Note, that immunohistochemical analysis highly correlates with counts obtained by FACS analysis. (**h**) Quantification of microglial proliferation with EdU assay by FACS analysis. Note that selective blockade of CSF1R reduces proliferation of microglial cells in SOD1^G93A^ mice at 16 weeks of age (n = 4 animals per group and time point). (**i**,**j**) Representative images of microglial proliferation by double immunofluorescence for EdU (red), Iba1 (green) and DAPI (blue) in the ventral horn of L4-L5 spinal cord tissue sections of (**i**) SOD1^G93A^ control and (**j**) treated with GW2580 at 16 weeks of age. Note that CSF1R blockade significantly reduces microglia proliferation but only at 16 weeks of age. Scale bars: (**d–f**) 200 μm; (**i**,**j**) 50 μm. *p < 0.05 compared to SOD1^G93A^ untreated, ^#^p < 0.05 compared to WT. Error bars indicate SEM.

**Figure 3:**
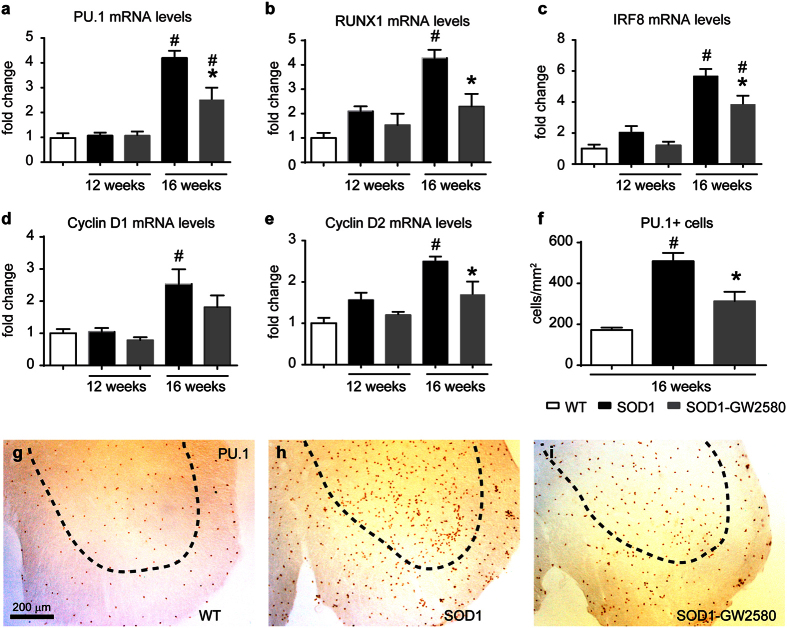
Effects of GW2580 treatment on CSF1R signalling in the spinal cord of SOD1^G93A^ mice. (**a**–**e**) Assessment of the mRNA levels of CSF1R transcription factors (**a**) PU.1, (**b**) RUNX1 and (**c**) IRF8 and cell cycle regulators (**d**) cyclin D2 and (**e**) D1 in the ventral lumbar spinal cord of SOD1^G93A^ mice. (**f**) Graph showing the reduction of PU.1+ cells in the ventral horn of spinal cords after GW2580 treatment in 16 weeks old SOD1^G93A^ mice. (**g**–**i**) Representative immunohistochemical images showing PU.1+ cells in the ventral horns of L4-L5 segments of WT (**g**), SOD1^G93A^ control (**h**) and GW2580-treated SOD1^G93A^ mice (**i**). G-I Scale bars 200 μm. *p < 0.05 compared to SOD1^G93A^, ^#^p < 0.05 compared to WT. (n = 3 WT; n = 4 for SOD1^G93A^ control and SOD1^G93A^ treated with GW2580 per time point). Error bars indicate SEM.

We have recently reported the importance of CSF1R pathway in inducing microgliosis in a mouse model of prion disease^17^. To determine whether CSF1-CSF1R signalling mediates microgliosis in ALS, we pharmacologically blocked CSF1R activation with GW2580, a selective CSF1R inhibitor^23^. FACS analysis of lumbar spinal cords of SOD1^G93A^ mice revealed that treatment with GW2580 reduced the number of microglial cells (CD45^low^, CD11b^+^) by ~30% at late, but not at earlier stages of the disease (Fig. 2a–c). Reduction of microgliosis by GW2580 treatment was further confirmed by immunohistochemical analysis of lumbar spinal cord of 16 weeks old SOD1^G93A^ mice (Fig. 2d–g). Attenuation of microgliosis by GW2580 was due to inhibition of microglia proliferation, since the number of EdU+ microglial cells was decreased by ~30% after treatment, as also revealed by FACS and histological analysis (Fig. 2h,j). Further evidence for the dependence of microgliosis on CSF1R signalling was observed: GW2580 treatment reduced the mRNA levels of three transcription factors that play a key role of microglia proliferation and lineage commitment, PU.1, IRF8, and RUNX1, and down-regulated the expression of the CSF1R downstream regulator of the cell cycle, cyclin D2, but not D1 (Fig. 3a–e). Histological studies revealed that PU.1+ microglial cells were reduced in the spinal cord of ALS mice after GW2580 treatment (Fig. 3f–i), corroborating our PCR data. Overall, these results demonstrate the importance of CSF1R activation in triggering microglial proliferation at late stages of the disease in the SOD1^G93A^ mouse.

### Effects of CSF1R inhibition on the neuroinflammatory response in SOD1^G93A^ mice

**Figure 4:**
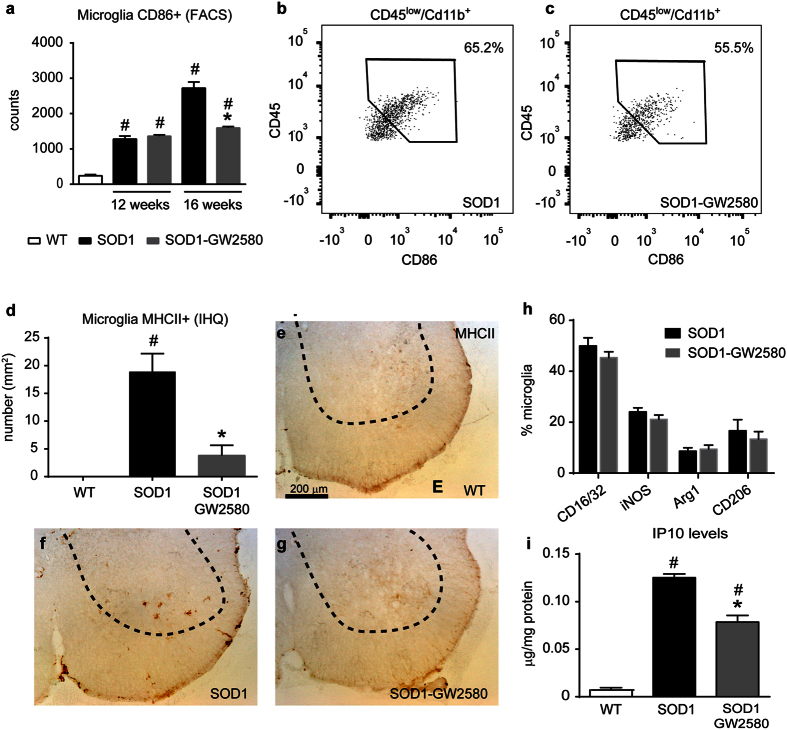
Effects of CSF1R blockade microglial cell activation and polarization in lumbar spinal cord of SOD1^G93A^ mice. (**a**) FACS analysis data showing CD86+ microglia in the lumbar spinal cord. Note that inhibition of CSF1R results in reduction of CD86+ microglial cells in SOD1^G93A^ mice at 16 weeks, but not at 12 weeks. (**b**,**c**) Representative dot plots showing CD86 expression in the gated microglia population (CD45^low^, CD11b^+^) of 16 weeks old SOD1^G93A^ control mice (**b**) and treated with GW2580 (**c**). Note the reduced counts of CD86+ microglia after the inhibition of CSF1R. (**d**–**g**) Quantification of MHCII immunostaining in the ventral horns of lumbar spinal cord tissue sections of 16 weeks old WT (**d**) SOD1^G93A^ control (**e**) and GW2580-treated SOD1^G93A^ (**g**) mice. (**h**) FACS analysis of different microglia activation markers from microglial cells from lumbar spinal cord at 16 weeks of age SOD1^G93A^ mice control or treated with GW2580. (n = 3 WT; n = 4 in SOD1^G93A^ mice untreated and treated with GW2580, per time point for each of the experiments). (**i**) Assessment of IP10 protein levels in the lumbar spinal cord of WT, SOD1^G93A^ control and treated with GW2580. *p < 0.05 compared to SOD1^G93A^, ^#^p < 0.05 compared to WT. Error bars indicate SEM.

We next assessed whether the inhibition of CSF1R activity modulated the inflammatory milieu in ALS. We first examined the effects of CSF1R on microglial cell activation and polarization. The expression of surface molecules related to antigen presentation is a hallmark of activated microglia^24^. Thus, we used CD86 and MHCII as markers for activated microglia^25^. FACS analysis revealed that GW2580 administration led to a significant reduction in the number of CD86+ activated microglial cells (Fig. 4a–c) and a reduction in MHCII+ microglia by the CSF1R inhibitor at the histological level (Fig. 4d–g), providing clear evidence that GW2580 attenuates activated microglia counts. However, reduction in CD86+ and MHCII+ cells is likely due to ability of the drug to hamper microglia proliferation since the proportion of these markers expressed in microglial cells is quite similar. FACS analysis revealed that CSF1R does not indiscriminately modulate microglia polarization in ALS, since the expression of CD16/32, iNOS, Arg1 and CD206 markers in microglial cells remained unaltered after GW2580 treatment (Fig. 4h). Similarly, we found that CSF1R inhibition had a minimal effect on cytokine production, since only the protein levels of one (IP10/CXCL10) out of the 11 cytokines evaluated were significantly reduced in the spinal cord homogenates of SOD1^G93A^ mice after GW2580 treatment (Fig. 4i).

### Selective inhibition of CSF1R slows the disease progression in SOD1^G93A^ mice

**Figure 5:**
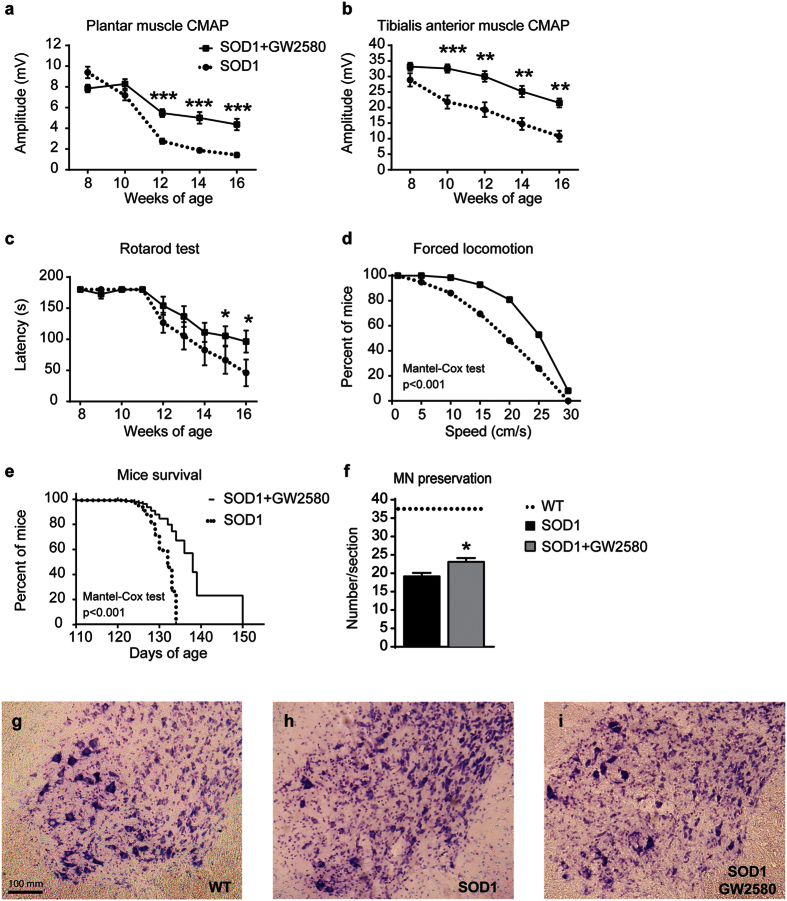
Administration of GW2580 preserves lower motoneuron function and extends SOD1^G93A^ mice survival. (**a**,**b**) Electrophysiological test showing preservation of the compound muscle action potential (CMAP) in both (**a**) plantar interossei and (**b**) tibialis anterior (TA) muscles (n = 18 control SOD1^G93A^ vs. 19 GW2580-treated SOD1^G93A^). (**c**) Treatment with GW2580 leads to significant preservation in functional outcomes assessed by rotarod (n = 10 per group). (**d**) Locomotor performance, evaluated on a treadmill, also reveals that GW2580-treated 16 weeks old mice are able to run at higher velocities than age-matched untreated SOD1^G93A^ mice (n = 10 per group). (**e**) GW2580 treatment significantly extends life span of female SOD1^G93A^ mice with a maximum of 150 days. (n = 10 per group). (**f**–**i**) Representative micrographs of lumbar spinal cord taken from WT (**g**) SOD1^G93A^ control (**h**) and SOD1^G93A^ treated GW2580 (**i**) showing motoneurons stained with cresyl violet; arrows indicate surviving motoneurons. (**f**) Graph showing quantification of motoneurons in the lumbar spinal cord. Lines indicate the average number of motoneurons in WT mice. (**g**–**i**) Scale bars 200 μm. *p < 0.05, **p < 0.01, ***p < 0.001 compared to SOD1^G93A^ untreated mice. Error bars indicate SEM.

We next assessed the contribution of CSF1R to the functional outcome in the SOD1^G93A^ mouse model of ALS. GW2580 administration led to a significant preservation of the amplitude of the TA and PL CMAPs starting at 10 and 12 weeks, respectively, which was sustained until the end of the follow up (16 weeks of age) (Fig. 5a,b). We also assessed the effects of GW2580 treatment on motor skills. Rotarod testing revealed that GW2580 did not delay the onset of the motor impairment but slowed its progression (Fig. 5c). Moreover, mice treated with GW2580 were able to run at significant higher speeds on a treadmill at 16 weeks of age (Fig. 5d). In line with the functional data, treatment with GW2580 led to significant extension in the survival of SOD1^G93A^ mice, increasing the maximal lifespan by 12% (Fig. 5e). Histopathological analysis of the lumbar spinal cord from SOD1^G93A^ mice at 16 weeks of age revealed that GW2580 increased significantly the number of surviving MN compared to control SOD1^G93A^ mice (Fig. 5f–i). These data provide clear evidence of the detrimental contribution of CSF1R activation in the CNS of ALS mice.

### CSF1R inhibition decreases the number of macrophages into the tibial nerve of SOD1^G93A^ mice

**Figure 6:**
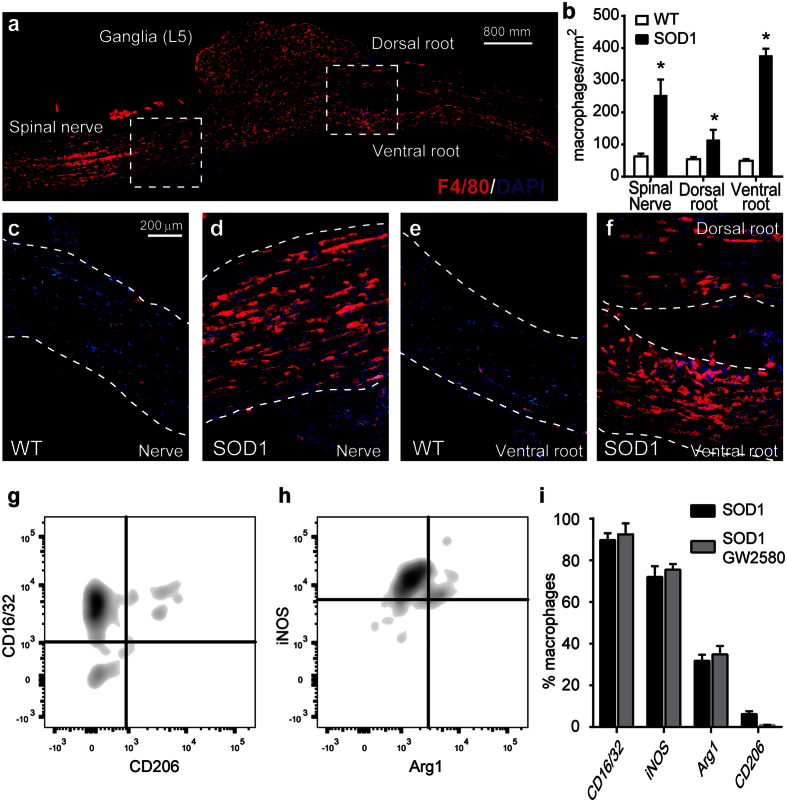
Macrophages infiltrate in the peripheral nerve of SOD1^G93A^ mice. (**a**) Representative image from L5 spinal nerve longitudinal section displaying the presence of macrophages in the nerve, as well as, in ventral and dorsal roots of SOD1^G93A^ mice at 16 weeks of age The area outlined in the boxes are shown in higher magnification in panels (**d**–**f**). (**b**) Quantification of macrophages in the L5 spinal nerve, dorsal and ventral roots of SOD1^G93A^ mice at 16 weeks of age (n = 4). (**c**–**f**) Representative images of L5 spinal nerve and ventral roots of WT and SOD1^G93A^ mice at 16 weeks of age. Note the macrophages are scarce in tissue sections from WT but abundant in those from ALS mice. (**g**–**i**) Evaluation by flow cytometry of CD16/32, iNOS, Arg1, and CD206 in macrophages present in the sciatic nerve of SOD1^G93A^ mice at 16 weeks of age untreated or treated with GW2580 (n = 3). *p < 0.05 vs WT.

**Figure 7:**
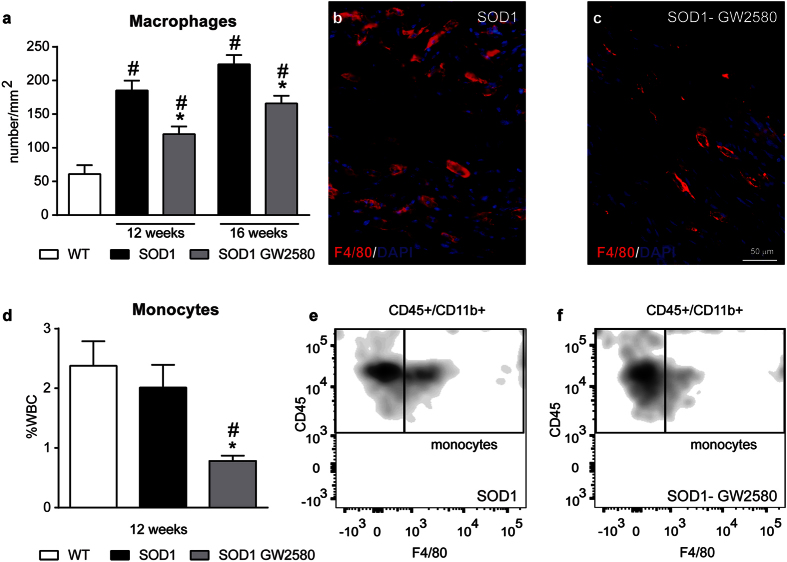
CSF1R attenuates infiltration of monocytes into tibial nerves of SOD1^G93A^ mice. (**a**) Quantification of F4/80+ cells in tibial nerve tissue sections of SOD1^G93A^ mice control or treated with GW2580 at the age of 12 and 16 weeks. (**b**,**c**) Representative images from tibial nerve longitudinal sections showing F4/80+ cells (red) inside nerve bundles in (**b**) SOD1^G93A^ control and (**c**) SOD1^G93A^ treated with GW2580. (**d**) Assessment by flow cytometry of circulating monocytes in both, GW2580-treated and control, SOD1^G93A^ mice. (**e**,**f**) Representative density plots showing blood monocytes (CD45+, CD11b high, F4/80+) in (**e**) control SOD1^G93A^ and (**f**) SOD1^G93A^ treated with GW2580. Note that CSF1R inhibition by GW2580 leads to monocytopenia. n = 4 per group, experiment and time. Scale bar 50 μm. *p < 0.05 vs. SOD1^G93A^ control mice; ^#^p < 0.05 compared to WT. Error bars indicate SEM. Scale bar 50 μm.

GW2580 treatment ameliorated disease progression in SOD1^G93A^ mice but the protective effects on muscle innervation were observed before the increase of CSF1 protein levels in the spinal cord, and consequently, prior to its effects on microgliosis. These findings suggest that activation of peripheral CSF1R might be responsible for the early muscle denervation. We therefore assessed whether this effects could be due to recruitment of macrophages into the sciatic nerve of SOD1^G93A^ mice. In agreement with a previous work^14^, we found that the presence of macrophages was markedly increased in the L5 spinal nerve of ALS mice at end-stage of the disease relative to WT controls. Macrophages were also abundant in the ventral roots, and to lower extent, in the dorsal roots (Fig. 6a–f). These macrophages showed predominant expression of CD16/32 and iNOS, whereas only a small subset of them displayed Arg1 and CD206 (Fig. 6g–i). Similar to that observed in microglia, GW2580 treatment did not change the expression of these markers in peripheral nerve macrophages (Fig. 6i). We then examined whether GW2580 treatment attenuated macrophage infiltration into the peripheral nerves of ALS mice prior to its anti-mitotic effects in microglia (12 weeks), as well as at end-stage of the pathology (16 weeks). We found, in comparison to control nerves, that macrophages were already abundant in the tibial nerve of SOD1^G93A^ mice at the clinical onset of the disease (12 weeks) that tended to increase in number over the course of the pathology (Fig. 7a), which is in agreement with a previous report^14^. Interestingly, GW2580 reduced macrophage counts in the tibial nerve of SOD1^G93A^ mice at 12 and 16 weeks (Fig. 7a–c), suggesting an important role of CSF1R for macrophage accumulation into peripheral nerves of ALS mice during early denervation stages. Since most macrophages in degenerating PNS derive from bloodstream in diverse conditions^26^,^27^ including in ALS^14^ it seems likely that reduction of PNS macrophages counts after GW2580 treatment is due to reduced invasion of monocytes. CSF1R has a key role in triggering hematopoietic stem cells to differentiate into monocytes, we thus assessed whether the reduced infiltration of macrophages into the nerve induced by GW2580 treatment of SOD1 mice was due to monocytopenia. FACS analysis of blood samples collected from 12 weeks old SOD1^G93A^ mice, the time point at which the reduction in nerve macrophages was already evident after GW2580 administration, revealed that the CSF1R inhibitor reduced the counts of circulating blood monocytes by about 2.5 fold (Fig. 7d–f). Overall, these findings highlight the importance of macrophage infiltration into the PNS in the pathophysiology of ALS.

## Discussion

In the present study we assessed the role of CSF1R in a mouse model of ALS. We determined that the expansion of microglial cells that occurs during the pathological course of ALS pathology in the SOD1^G93A^ model is largely due to cell proliferation. We also observed that selective blockade of CSF1R inhibited microglial cell proliferation, and consequently, microgliosis. Treatment with GW2580 exerted beneficial effects in ALS pathology, slowing disease progression and extending mice survival. The protective actions of GW2580 are partially explained by its effect on microglial CSF1R, since the beneficial effects of GW2580 on motor conduction tests were achieved prior to its actions on microglia proliferation. Interestingly, we found that GW2580 treatment reduced the number of macrophages recruited to the peripheral nerves of SOD1^G93A^ mice before microgliosis occurred, which was likely due to the monocytopenia induced by CSF1R inhibition. Overall, these results demonstrate the importance of central and peripheral activation of CSF1R signalling in ALS pathophysiology, and support the strategies targeting CSF1R activation as possible therapeutic approaches.

Inflammation is a hallmark of chronic neurodegenerative diseases, including ALS. Two immune cell subsets predominate in two different compartments of the nervous system in ALS disease: microglia in the CNS and macrophages in the PNS. Microglia contribute to neurodegeneration in numerous neurological conditions^16^,^17^,^28^,^29^, however, their role in ALS is currently under debate due to conflicting results. Macrophages play divergent roles in several PNS and CNS disorders^26^,^30^, however, whether they exert detrimental, helpful or even neutral effects in ALS has not been elucidated yet.

In agreement with previous studies^31^,^32^, we show that the prominent microgliosis that occurs in ALS is due to increased proliferative activity of resident microglial cells. Although the exact mechanisms that trigger the mitotic programme in microglia are not fully understood, recent works suggest that CSF1 signalling via CSF1R mediates the proliferation of microglia in various CNS disorders^17^,^33^,^34^. In the present experiments, we show that CSF1, but not IL-34, is up-regulated in the spinal cord of SOD1^G93A^ mice and that the treatment with GW2580, a selective CSFR1 inhibitor, attenuates microglial cell expansion and slows the progression of ALS disease. The reduction of microglial cells by GW2580 is due to inhibition of cell proliferation based on our analysis on EdU incorporation and gene expression analysis, highlighting the importance of CSF1R activation in microglia proliferation in ALS. Interestingly, GW2580 treatment led to significant sparing of lumbar MNs, suggesting that microgliosis induced by CSF1R signalling contributes to neurodegeneration in this ALS mouse model.

The inhibitory effects of GW2580 on microgliosis were only observed at late stages of the disease in SOD1^G93A^ mice, which is due to the lack of CSF1 induction in early phases of the pathology. However, in agreement with others^14^,^18^ we found that microgliosis was evident at early stages of the disease in this ALS mouse model. This data therefore suggest that there are various mediators involved in microglia proliferation during the course of ALS pathology, CSF1R signalling is a key player at late but not at early stages of the disease. Indeed a recent study reveals that IL-1R signalling has a key function in microglial cell proliferation following microglia ablation^35^, which may explain the attenuated microgliosis observed in ALS mice treated with IL-1ra or lacking IL-1β expression^36^. The fact that CSF1R mediates microglia proliferation only at late stages of ALS disease is important in the present study, since the beneficial effects of GW2580 on motor function were already evident at the electrophysiological level from week 10–12, when CSF1 was not yet induced in the CNS. This observation suggests that although microgliosis triggered by CSF1R contributes to ALS disease, the early protective actions of GW2580 on muscle denervation are not mediated by microglial CSF1R but by peripheral CSF1R. Supporting these findings, a previous study reveals that systemic administration of CSF1 in another mouse model of ALS (SOD1^G37R^ mouse), which leads to over-activation of peripheral CSF1R, accelerated disease progression but not MN death, highlighting the importance of peripheral CSF1R in ALS pathology^18^.

Besides microglial cells, circulating monocytes show constitutive expression of CSF1R^37^. Although monocytes do not migrate into the spinal cord in ALS mice^31^,^38^, there is robust presence of macrophages in the peripheral nerves of ALS mice prior to the clinical onset of the disease^14^,^15^. Indeed, PNS macrophages and microglia show similar dynamics of expansion in ALS^14^. In contrast to microglia, macrophages found within the nerves of ALS mice are derived from circulation (~75%), as shown by experiments where GFP bone marrow cells were transplanted into irradiated ALS mice^14^. Indeed, the expression of CCL2, one of the main chemoattractants for monocytes, in the nerve of ALS mice correlates with the accumulation of macrophages^14^. This is likely due to early distal axonal degeneration that occurs in the nerves of ALS mice and patients before the death of MN^39^,^40^, which may trigger macrophage migration into the nerve. Although macrophages are crucial for proper axonal regeneration after PNS damage, they can also mediate detrimental effects in different PNS conditions^26^. Indeed, SOD1^G93A^ macrophages exert toxic effects on primary neuronal cell cultures^41^, and chimeric mice have shown that myeloid cells express mutant SOD1 and that they are harmful to neurons^42^,^43^. These reports therefore suggest that macrophages expressing mutant SOD1 may not mediate repair in ALS. Indeed, here we show that macrophages that infiltrate into the sciatic nerve of ALS mice display a pro-inflammatory phenotype, associated with tissue damage^26^. Herein we found that GW2580 reduced the numbers of macrophages into the nerves of SOD1^G93A^ mice. Since PNS macrophages are mostly recruited from circulation in ALS^14^, this data suggests that the entrance of monocytes into the PNS is attenuated when CSF1R signalling is blocked. In line with our results, systemic administration of CSF1 increased the numbers of macrophages in the sciatic nerves of ALS mice^18^. Similarly, a recent report indicates the CSF1R has a key role for the infiltration of macrophage in the sciatic nerve of a Charcot Marie Tooth mouse model, where they produce adverse effects^44^. The reduced migration of monocytes into the tissues after treatment with GW2580 is likely due to the importance of CSF1R in promoting the differentiation of bone marrow cells into monocytes^45^ since we observed that the CSF1R blockade led to monocytopenia in the ALS mice. However, we cannot rule out the possibility that the disruption of CSF1R signalling could also interfere with monocyte chemotaxis and/or with the proliferation of resident macrophages. Interestingly, the reduction in the number of PNS macrophages observed in the SOD1^G93A^ mice treated with GW2580 prior to the clinical onset of the disease correlated with the greater preservation of the electrophysiological responses. These results indicate that, besides the harmful effects of microglia in ALS, macrophages have also significant impact on disease progression by acting in the PNS. This is a new inroad into the functions of macrophages in the pathophysiology of ALS since the therapeutic effects of multiple anti-inflammatory approaches in ALS have been exclusively attributed to their ability to interfere with microgliosis, but not with PNS macrophages.

Despite GW2580 attenuated microgliosis and macrophage counts in the peripheral nerves of SOD1^G93A^ mice, its administration only increased the maximal lifespan by 12%. This modest effect on mouse survival can be explained, in part, by the lower inhibitory activity of GW2580 on CSF1R as compared to novel compounds such as PLX3397. However, whereas GW2580 shows high selectivity for CSF1R^23^, PLX3397 has also potent inhibitory effects on c-Kit, FTL3 and PDGFR, and thus, it does not allows one to precisely dissect out the role of CSF1R. It is likely that the generation of more potent and selective CSF1R inhibitors will result in greater beneficial effects^46^.

In summary MN pathology in ALS disease, which begins with distal axonal pathology and proceeds in a dying back pattern, is likely to be the main trigger of inflammation in the PNS and in the CNS, which in turn contributes to the course of the pathology by accelerating muscle denervation and MN degeneration in a positive feedback loop. Our work reveals that CSF1R signalling has a crucial role in modulating the immune response in the CNS and PNS in SOD1^G93A^ mouse model of ALS. Although further studies are needed to elucidate the exact molecular mechanisms underlying the combined deleterious actions of macrophages and microglia in ALS, our data suggest that CSF1R signalling could be a novel therapeutic target in ALS.

## Additional Information

**How to cite this article**: Martínez-Muriana, A. *et al.* CSF1R blockade slows the progression of amyotrophic lateral sclerosis by reducing microgliosis and invasion of macrophages into peripheral nerves. *Sci. Rep.* **6**, 25663; doi: 10.1038/srep25663 (2016).
